# Profile Pictures in the Digital World: Self-Photographs Predict Better Life Satisfaction

**DOI:** 10.3390/ijerph18126667

**Published:** 2021-06-21

**Authors:** Kalai Hung, Naomi A. Lee, Kaiping Peng, Jie Sui

**Affiliations:** 1 Department of Psychology, Tsinghua University, Beijing 100084, China; pengkp@mail.tsinghua.edu.cn; 2 School of Psychology, University of Aberdeen, King’s College, Old Aberdeen AB24 3FX, UK; n.lee.20@abdn.ac.uk (N.A.L.); jie.sui@abdn.ac.uk (J.S.)

**Keywords:** profile picture, online-offline self-consistency, personality, gender, life satisfaction, self-representation, digital health

## Abstract

Profiles pictures as online identities represent an extension of the user’s self in the digital world. Changes in self-representation are responsible for reduced well-being in individuals in the offline world. However, whether profile picture selection predicts the well-being of internet users is unknown. To address this question, we tested the relationship between the type of profile picture (e.g., self-photographs or other pictures) used on social media and the life satisfaction of internet users, accounting for gender and personality traits that have been thought to relate to the selection of profile pictures. The results showed that individuals using self-photographs as profiles reported a higher level of life satisfaction compared to individuals using other pictures as profiles. This effect was influenced by gender, openness, and extraversion. Hierarchical regression and moderation analyses revealed that openness and profile type interacted to predict life satisfaction in women, while openness and profile picture independently predicted life satisfaction in men. Furthermore, extraversion directly predicted life satisfaction in both men and women. These results indicate that the consistency between one’s online and offline self-representation may characterize internet users’ well-being, with potential implications for digital wellness.

## 1. Introduction

Boundaries between the online and offline worlds are becoming increasingly blurred in today’s world. Belk (2016) suggested that there was a consistency of identity construction from offline to online interactions [1]. A representative example of online self-image management is the selection of profile picture on social networking sites. A profile picture refers to a specific picture that individuals use in online social networking to represent themselves, which can be any type of picture, and it reflects how individuals view and shape their online self-representation [2]. Changes in self-representation, on the other hand, have been linked to a variety of mental illnesses in the offline world [3,4]. Therefore, the phototype that individuals choose as profiles to present themselves online (e.g., using self-photographs or other pictures) may relate to mental health such as subjective well-being, because this social networking behavior reflects the consistency of online and offline self-representation. The aim of the current study was to examine whether this was the case by focusing on the relationship between the use of online identity and life satisfaction.

The profile picture is the most visible user-generated characteristic in the online world as it appears in almost every online interaction such as every response message [5,6]. Among different types of profile picture, self-photographs are one of the most common and unique types [7]. A photograph of the self means a picture that includes only the self, regardless of whether this picture was taken by others or by themselves (i.e., selfie) [8]. Several studies have explored the relationship between self-photographs as profile pictures and well-being. Mills et al. (2018) [9] found women who uploaded their self-photographs on social media were more likely to feel nervous, insecure, and unattractive compared to those who did not. The authors claimed that this social networking behavior elicited the feeling of self-awareness and the fear of others’ negative assessment. Likewise, it has been argued that taking and sharing self-photographs can result in greater social sensitivity and lower self-esteem [10]. However, recent work has demonstrated a positive effect of the use of self-photograph in social media on well-being. Yue et al. (2017) [11] found that taking self-photographs as profiles were positively correlated with the level of life satisfaction. The authors have argued that by taking an enhanced self-photograph as a profile picture, individuals are likely to get more positive feedback through social media, thus improving their satisfaction with interpersonal relationships through the digital world. One way to disentangle these conflicting results is to consider factors that affect the selection of profile pictures. From previous studies, two critical candidates are personality and gender [12].

One key factor that influences the selection of online profile pictures is personality traits, particularly two dimensions of extraversion and openness. It has been found that there is a positive link between the frequency of self-photograph posting and sharing in social media and the level of extraversion [13]. Individuals high in extraversion tend to more frequently use groupies (e.g., photographs of self pictured with others) as profiles, and those individuals also have a large social network in the offline world [14]. The authors claimed that extraverts hold a high level of online–offline consistency to maintain their social connections with others [14]. Liu et al. (2016) [15] used visual features (e.g., color, face, and emotion) of Twitter user profile pictures to predict personality traits, and they reported that people high in openness use more non-self facial images as their profile pictures (but see [16]). These previous findings are consistent with the view that extraversion and openness can be used to predict personality traits [17]. Furthermore, there is evidence that extraversion and openness are the main predictors of well-being. A meta-analysis study revealed that both openness to experience and extraversion were positively related to happiness, positive affect, and quality of life [18].

In addition to personality traits, gender is another important factor influencing online self-representation [19]. For example, women are reported to post more self-photographs on social media than men [20], and they tend to devote more time to managing their online profiles and share more photos than men, which may relate to women’s larger network of strong ties in the offline world [21]. In addition, women are more likely to believe their profile pictures accurately reflect their personalities [22]. On the other hand, it has been found that there was a positive correlation between histrionic personality and the number of self-photographs in men, but the effect was not observed in women [23]. Reed and Saunders (2020) [24] have argued that the gender differences in profile pictures could be attributed to the need for mate selection in an evolutionary context in which women are more willing to express themselves by posting the self-photograph.

In summary, online self-representation is a crucial facet of subjective well-being in internet users. However, less is known about the exact association between online self-representation and well-being and how their relationship is influenced by vital individual factors, such as gender and personality. The profile picture is the most prominent self-representation of internet users. Therefore, the primary aim of the present study was to examine the relationship between life satisfaction and online self-representation in internet users using profile pictures (self-photographs vs. other pictures) and to investigate whether and how gender and personality (i.e., two dimensions of extraversion and openness) influence this relationship. Do extraversion and openness, together with the selection of profile pictures in the digital world, influence life satisfaction in internet users? Is there a gender difference in such an effect? Based on previous research that the levels of consistency in offline–online self-representation index the subjective well-being in internet users [25], we predicted that individuals using self-photographs as profile pictures, in comparison to individuals using other pictures as profiles, would show a higher degree of life satisfaction. In addition, due to mixed results regarding the role of gender in the relationship between well-being and profile pictures, and no previous studies investigating the associations between profile pictures, personality, and life satisfaction, we had no specific hypotheses regarding how gender and personality traits (i.e., openness and extraversion) would influence the relationship between profile picture usage and the life satisfaction of internet users.

## 2. Materials and Methods

### 2.1. Participants

A total of 302 participants took part in the experiment (136 males and 166 females, mean age = 25.2 years, *SD* = 3.84, ranging from 18 to 34 years old). Two hundred and four participants completed the task in the lab and were paid with either course credits or monetary rewards. The other 98 subjects were recruited through an online experiment platform (Prolific) and were paid monetarily through the website. Based on our experimental hypothesis, there are three factors to be examined, namely the type of profile pictures, gender, and personality traits (i.e., openness or extraversion). The experiment was reviewed and approved by the local ethics committee. Informed consent was obtained from the participants prior to the experiment.

### 2.2. Measures

#### 2.2.1. Life Satisfaction

To evaluate participants’ subjective life satisfaction, we used a five-item life satisfaction scale [26]. Participants were asked to indicate their level of agreement with items on a seven-point Likert scale (e.g., “I am satisfied with my life”), with 1 = strongly disagree and 7 = strongly agree (Cronbach’s α = 0.847). In line with Diener et al. (1985) [26], we used the sum score of all items as a continuous variable. The mean score across participants was 20.5, suggesting a moderate level of subjective life satisfaction.

#### 2.2.2. Openness and Extraversion

The Big Five Inventory (BFI) [27] was used to measure participants’ personality traits. The BFI is a 44-item questionnaire measuring five personality traits. All items were measured on a five-point Likert scale (1 = strongly disagree; 5 = strongly agree). Based on previous studies that assert that openness and extraversion influence social networking behaviors, we focused on the two dimensions for data analysis [19]. The reliability for each dimension in our sample was as follows: extraversion (8 items; Cronbach’s α = 0.80) and openness (10 items; α = 0.71).

#### 2.2.3. Type of Profile Pictures

Participants were asked to indicate and describe the type of profile picture that they favored on their most commonly used social networking platform (e.g., whether their profile image was a self-photograph or not). The majority (67.5%) of the participants reported that their profile pictures were online profiles on the WeChat platform, and the other 32.5% were on Facebook. The social media platform type was used as a controlling factor in data analyses. In this study, we classified all the images into two categories, self-photograph and other pictures. We defined the self-photograph as a photograph that includes the self alone, regardless of whether this picture was taken by others or by the self (e.g., selfie) [28], and other image types (e.g., pets, cartoon, landscapes, etc.) are collectively referred to as other pictures. There were 101 participants (33.4%) who used a self-photograph and 201 who used other pictures. 

### 2.3. Data Analysis

Life satisfaction scores were taken as the dependent variable, while gender, profile type, and openness (or extraversion) were treated as independent variables. We first conducted correlation analysis to explore the relationships between the dependent and independent variables. Based on the correlation results, the key predictors were used in two separate hierarchical regression models for openness and extraversion to calculate the contribution of each predictor to the dependent variable, life satisfaction. Finally, moderation analyses were performed when significant interactions between predictors were observed in the hierarchical regression models.

## 3. Results

We conducted correlation analyses to explore the relationships between life satisfaction, profile picture type, and personality (openness and extraversion). Table 1 displays the means, standard deviations, and correlations among variables. Life satisfaction was positively correlated with profile picture types (*r* = 0.167), extraversion (*r* = 0.426), and openness (*r* = 0.141). People that used a self-photograph as a social networking profile picture perceived higher subjective life satisfaction than other picture users. We found there was no significant correlation between profile type and gender, openness, or extraversion.

### 3.1. Openness

Based on the correlation analyses, key predictors were used in the hierarchical regression models to calculate the impact of each predictor on life satisfaction. We entered age and social media platform in Model 1, adjusting for age and social media platform. All main predictors, gender, openness, and profile picture type were entered in Model 2. We also tested all two-way interactions (openness × gender, openness × profile picture type, gender ×profile picture type) in Model 3. Finally, the moderating effects were tested by adding a three-way interaction (openness × gender × profile picture type) in Model 4.

All the results of hierarchical regression models are presented in Table 2. As we predicted, the profile picture type was positively correlated with life satisfaction, people who use self-photographs as profile pictures perceived higher life satisfaction (*β* = 0.288, *p* = 0.015). As shown in Model 3, all the two-way interactions were not significant. Model 4 was significant, *F*(8, 293)= 3.899, *p* < 0.001, with an adjusted *R*^2^ = 0.080. Notably, the three-way interaction term (openness × gender × profile picture type) was significant (*β* = 0.618, *p* = 0.007), indicating that gender moderates the interaction effect of openness and profile picture on life satisfaction. 

**Figure 1:**
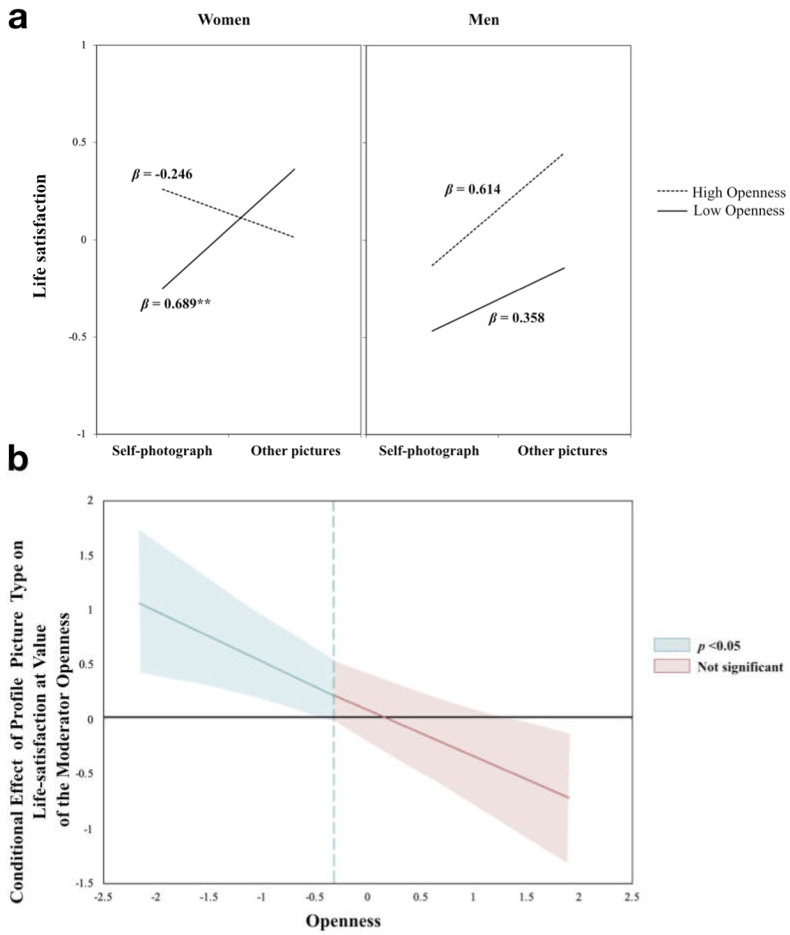
(**a**) Interaction of openness and profile type on life satisfaction. (**b**) Region of significance (95% confidence interval) for the conditional relation between openness and profile type on life satisfaction. *Note.* The dashed vertical line denotes the turning point from non-significance to significance of the effect of openness.

To deconstruct these interaction effects in the hierarchical regression analyses, a simple slope test was conducted using the Johnson–Neyman technique [29] at 1 SD above or below the mean of openness. For women, the analysis showed that individuals whose openness was 1 SD below the mean with self-photographs as the profile picture were more likely to have a higher subjective life satisfaction (*β* = 0.689, *p*= 0.002); in contrast, no such effect was observed for women with above-average openness (*β* = −0.246, *p* = 0.235). In summary, for women low in openness, the use of a self-photograph as their profile image was related to increased life satisfaction, while for women high in openness, the profile picture type did not predict life satisfaction (see Figure 1a). The two slopes differed significantly from each other (*t* = −5.797, *p* < 0.001).

The analysis on men showed a direct effect of profile type on life satisfaction (*β* = 0.531, *p* = 0.007). Men using self-photographs as profile picture perceived higher life satisfaction than those using other pictures. Openness was not a significant predictor for men in life satisfaction (*β* = 0.193, *p* = 0.069). The moderation analysis failed to show a moderation effect of openness on the association between profile type and life satisfaction (*β* = 0.121, *p* = 0.505) (see Figure 1b). These two slopes did not differ significantly from each other (*t*= 0.838, *p* = 0.403).

### 3.2. Extraversion

Then, we tested the influence of profile type and extraversion on life satisfaction (Table 3). The hierarchical regression results indicated that when controlling for age and social media platforms, extraversion (*β* = 0.517, *p* < 0.001) was a strong predictor of life satisfaction, but this was not the case for gender (*β* = −0.109, *p* = 0.248) and type of profile pictures (*β* = 0.150, *p* = 0.154). All two-way and three-way interaction terms in Models 3 and 4 failed to predict life satisfaction, indicating that there was no moderation effect between extraversion, gender, and type of profile picture. 

## 4. Discussion

The present study tested whether the type of profile pictures–online self-portrayal predicted the subjective well-being (i.e., life satisfaction) of internet users and whether the effect was moderated by gender and the two dimensions of personality (i.e., openness and extraversion). We found that profile image type predicted life satisfaction. In addition, men using self-photographs as profile images tended to have greater life satisfaction than those using other pictures. There was no direct relationship between profile type and life satisfaction in women, but openness moderated the association between profile type and life satisfaction. Women with lower openness who used self-photographs as profile pictures reported higher life satisfaction. Furthermore, extraversion was a direct positive predictor of life satisfaction in both men and women.

As hypothesized, individuals who used a self-photograph as a profile picture on social media reported better life satisfaction, compared to those using other pictures as profiles. This finding suggests that individuals who use self-photographs as their online self-representation may hold a high level of consistency in offline–online self-representation, which is a crucial facet of subjective well-being in internet users. The result adds to evidence suggesting that a higher level of online and offline self-integration is associated with better mental health [25]. Psychiatric work has well documented that changes in self-representation are associated with reduced well-being and several mental disorders (e.g., depression and schizophrenia) [3]. Although there was no direct evidence for the relationship between mental health issues and the online-offline self-consistency, the current findings support the theoretical view that changes in self-representation (e.g., negative self-schema) are risk factors for mental disorders [30]. Our results indicate the importance of understanding the links between digital wellness and online–offline self-consistency in internet users, with potential implications that enhancing online–offline self-consistency may help to reduce negative influence on well-being.

In addition, the predictive ability of profile types on life satisfaction was particularly notable for men. These results were consistent with past research suggesting that men tend to use more authentic self-photographs as profiles than women [24], reflecting that the use of profile pictures in men may directly predict the level of life satisfaction. There was no direct association between profile type and life satisfaction in women. Such finding suggests that women’s cases may be more complicated. Women are more impacted by the negative impact of social media [4]. Yin et al. (2019) [31] performed a meta-analysis to test the relationship between social networking behaviors and mental health, and they found a negative correlation between social networking usage and mental health in female users. Furthermore, they suggested that there may be complex factors that moderate the relationship between internet use and mental health in women. 

There was a significant moderation effect of openness to experience on the association between profile picture type and life satisfaction. Women with low openness who used self-photograph profile pictures were more likely to report higher life satisfaction. From an evolutionary perspective, this result may reflect that women with lower levels of openness to experience express their ideal self through more selfie-related behaviors to enhance mating opportunities [12]. Posting and sharing self-photographs on social media represent an adapted mating strategy for successful mating, thus enhancing subjective well-being. In women with higher openness, on the other hand, openness did not modulate the linkage between profile type and life satisfaction. This suggests that women with a higher level of openness to experience in the offline world may focus on more actual activities in the offline world, so their mating choice may be more diverse. Thus, it is not surprising that there was no linkage between profile picture usage (e.g., self-photograph) and life satisfaction for women with higher openness. Nonetheless, there was no moderation effect of extraversion on the relationship between profile picture types and life satisfaction in women (and men). Extraversion directly predicted individuals’ life satisfaction for both men and women. This finding is consistent with previous findings that the extraversion trait is a robust predictor of subjective well-being [32].

### 4.1. Implications

Previous studies have addressed that changes in self-representation relate to adverse mental health problems [3]. Here, we explored this relationship in a positive way. We investigated the consistency of the online and offline self by comparing individuals with self-photographs as profiles to those using other pictures. We found that this dichotomous variable is valid to predict life satisfaction. However, changes in self-representation should be considered a continuous variable. Previous studies have demonstrated that profile pictures can be quantified using fine-grained analyses to detect different features of online self-representation, which provide a powerful measurement to predict personality traits [17,33]. Therefore, future studies investigating the relations between profile pictures using these in-depth analyses, personality, and life satisfaction would provide insights into the roles of online self-representation in the subjective well-being of internet users. In addition, the relationship between online behaviors and mental health has become of particular concern in recent years as users’ online activities have increased over time. It has been suggested that users’ online behavior can serve as a valuable source of data for public health surveillance [34]. Therefore, one practical implication of the present findings is that using self-photographs as profiles on social media may be a notable feature of the internet users with better mental health status. However, as noted by Saura and colleagues [6,35], user data should be collected and analyzed in a way that does not invade the privacy of users and follow social media ethics. 

### 4.2. Limitations and Future Research

There are several aspects on which future studies might improve and expand. First, as different social media platforms have different users [11], in-depth analysis with large samples may be fruitful to characterizing how online self-representation predicts subjective well-being in different platform users. Second, in the present study, we classified all non-self-photographs into the type of other pictures. It is worth further investigating the impact of different types of other profile images on life satisfaction in internet users. For example, the close others’ picture (e.g., a picture with families or friends) is related to a social aspect of the self and indicates one’s offline social network. Third, it is likely that the degree of selfie editing reflects life satisfaction, which requires fine-grained analyses in a large sample in the future. Finally, age is another factor that may influence the relationship between online–offline self-consistency and mental health [20,36]. The present study only tested young adults between 18 and 35 years old. Future work may validate the current findings using a life-course approach [37].

## 5. Conclusions

The type of profile images on social media can predict life satisfaction, suggesting that internet users with a well-unified online and offline self have greater overall life satisfaction. The relationship between profile type and life satisfaction was moderated by openness and gender. Extraversion independently predicted life satisfaction in both men and women. These results indicate that self-consistency in the online and offline world can be used to predict internet users’ well-being with potential implications for digital wellness.

## Figures and Tables

**Table 1:** Descriptive statistics and Pearson correlations among variables (*N* = 302).

Variables	1	2	3	4	5
1. Life-satisfaction	—				
2. Gender ^a, c^	−0.108	—			
3. Extraversion	0.426 ***	−0.055	—		
4. Openness	0.141 *	0.05	0.286 ***	—	
5. Type of profiles ^b, c^	0.167 **	−0.091	−0.017	0.006	
*M*	20.2	45%	3.28	3.49	33.4%
*SD*	6.28	N/A	0.925	0.6	N/A

*Note:* Gender and Type of profiles are binary variables, we coded ^a^ 1 = male, 0 = female; ^b^ 1 = self-photographs, 0 = nonactual self-images; ^c^ means the percentage of the amount; * *p* < 0.05, ** *p* < 0.01, *** *p* < 0.001.

**Table 2:** Hierarchical multiple regression models for openness predicting life satisfaction.

Predictor	Model 1	Model 2	Model 3	Model 4
Control				
Age	0.096	0.092	0.095	0.110
Platform	−0.068	0.044	0.087	0.143
Main effects				
Gender		−0.202	−0.302 *	−0.313 *
Openness		0.137 *	0.158	0.280 **
Profile picture		0.288 *	0.203	0.206
Two-way interactions				
Openness × gender			0.130	−0.101
Openness × profile picture type			−0.199	−0.485 **
Gender × profile picture type			0.085	0.096
Three-way interactions				
Openness ×gender × profile picture type				0.618 **
Adjusted *R*^2^	0.008	0.050	0.060	0.080
*R*^2^ change	0.015	0.051 **	0.019	0.023

*Note.* * *p* < 0.05, ** *p* < 0.01, *** *p* < 0.001; unstandardized coefficients are shown.

**Table 3:** Hierarchical multiple regression models for extraversion predicting life satisfaction.

Predictor	Model 1	Model 2	Model 3	Model 4
Control				
Age	0.096	−0.006	−0.002	0.002
Platform	−0.068	−0.572 ***	−0.567 ***	−0.563
Main effects				
Gender		−0.109	−0.561	−0.116
Extraversion		0.517 ***	0.459 ***	0.528 ***
Profile picture		0.150	0.159	0.849
Two-way interactions				
Extraversion × gender			0.218	−0.018
Extraversion × profile picture type			−0.033	−0.375
Gender × profile picture type			0.043	−0.403
Three-way interactions				
Extraversion × gender × profile picture type				0.469
Adjusted *R*^2^	0.008	0.261	0.259	0.265
*R*^2^ change	0.015	0.259 ***	0.005	0.009

*Note.* * *p* < 0.05, ** *p* < 0.01, *** *p* < 0.001; unstandardized coefficients are shown.

## Data Availability

The data used to support the findings of this study are available from the corresponding author upon request.
